# Cis‐regulatory and long noncoding RNA alterations in breast cancer – current insights, biomarker utility, and the critical need for functional validation

**DOI:** 10.1002/1878-0261.70157

**Published:** 2025-11-13

**Authors:** Arnau Cuy Saqués, Aracele Martinez‐Mendez, John Crown, Alex Eustace

**Affiliations:** ^1^ Life Science Institute Dublin City University Ireland; ^2^ Medical Oncology Department St Vincent's University Hospital Dublin Ireland; ^3^ School of Biotechnology Dublin City University Ireland

**Keywords:** biomarker discovery, breast cancer, enhancer mutations, long‐noncoding RNAs, noncoding genome, promoter mutations

## Abstract

Breast cancer's global prevalence underscores a critical need for novel biomarkers to guide treatment and improve patient outcomes. Biomarker discovery historically focused on mutations in protein coding regions, comprising merely 1% of the genome. However, with advances in whole‐genome sequencing, the functional significance of the noncoding genome—comprising the remaining 99%—has become increasingly evident. Noncoding regions play a vital role in regulating gene expression, and mutations within these regions have been associated with cancer risk, progression, and treatment response. This Review compiles and synthesizes current knowledge on cis‐regulatory alterations (promoters/enhancers) and long noncoding RNAs (lncRNAs) in breast cancer. Key examples include promoter mutations [e.g., rs2279744 (Mouse double minute 2 homolog gene; *MDM2*)], enhancer mutations [e.g., rs4784227 (thymocyte selection‐associated high mobility group box family member 3 gene; *TOX3*)], and lncRNAs [e.g., HOX transcript antisense intergenic RNA (*HOTAIR*)] linked to progression, metastasis, and poor survival. Integrating preclinical (*in vitro*, *in vivo*) and clinical findings, we emphasize the biomarker and therapeutic potential of these noncoding alterations. This Review also critically identifies the pressing need for more specific functional validation studies to fully elucidate their mechanistic roles. This emerging field offers promising opportunities to advance personalized medicine and refine prognostic/predictive strategies for breast cancer patients.

## Graphical abstract

The noncoding region of the genome plays a key role in regulating gene expression, and mutations within these regions are capable of altering it. Researchers have identified multiple functional noncoding mutations associated with increased cancer risk in the genome of breast cancer patients. However, further functional studies are needed to fully understand their role in cancer progression.

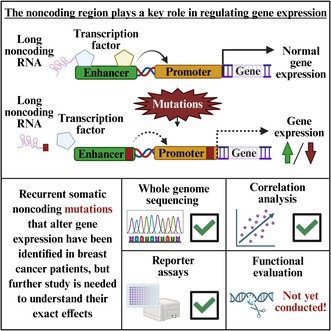

**Abbreviations**
BC: breast cancer
BRCA1: breast cancer gene 1
CBP: CREB‐binding protein
CREs: Cis‐regulatory elements
CTCF: CCCTC‐binding factor
DANCR: differentiation antagonizing non‐protein coding RNA
ER: estrogen receptor
ESR1: estrogen receptor 1 gene
ESR2: estrogen receptor 2
ETS: E‐twenty‐six
FNTB: farnesyltransferase subunit β
FOXA1: forkhead box protein A1
H19: lncRNA H19
HER2: human epidermal growth factor receptor 2
HOTAIR: HOX transcript antisense intergenic RNA
lncRNA: long noncoding RNAs
MALAT1: metastasis associated lung adenocarcinoma transcript 1
MDM2: mouse double minute 2 homolog
NEAT1: nuclear paraspeckle assembly transcript 1
OAS1: 2′‐5′‐oligoadenylate synthetase 1
RMRP: RNA component of mitochondrial RNA processing endoribonuclease
SNPs: single nucleotide polymorphisms
TERT: telomerase reverse transcriptase
TINCR: tissue differentiation‐inducing non‐protein coding RNA
TNBC: triple negative breast cancer
TNFSF10: tumor necrosis factor superfamily member 10
TOX3: thymocyte selection associated with high mobility group box family member 3
TRAIL: tumor necrosis factor‐related apoptosis‐inducing ligand
WGS: whole‐genome sequencing

## 1 Introduction: The emerging significance of the noncoding genome in breast cancer

Breast cancer (BC) is the most commonly diagnosed cancer and the leading cause of cancer death in women worldwide [1]. It is a heterogeneous disease, where a high degree of variety is observed in the genetic, epigenetic, and transcriptomic profile between different BCs, and it can even occur within the same tumor [2]. As coding mutations have the ability to impact disease progression and patient prognosis through changes in protein functionality, most research to date has been focused on areas of the genome that code for proteins [3]. However, by searching for driver mutations in the coding region of the genome, researchers were only studying 1% of the total DNA. The ENCODE project observed that around 80% of the genome is comprised of elements involved in the regulation of gene expression, thus highlighting the importance of studying the DNA regions outside of protein coding genes [4].

### 1.1 Study of the noncoding genome

One of the main reasons that the study of the noncoding genome in cancer has increased has been due to improvements in next‐generation sequencing technologies [5, 6]. Traditionally, researchers conducted genome‐wide association studies (GWAS) to identify the presence of preselected common genetic variants, and then associated them with the development of cancer or disease. However, GWAS is limited, by knowing in advance, which regions of the genome are going to be sequenced, so it fails to simultaneously study the full mutational profile of the noncoding genome [7]. Nowadays, the most common method to identify novel noncoding mutations (NCMs) consists of whole‐genome sequencing (WGS), which is based on the fragmentation of the DNA for sequencing and later mapping by homology. There are two main types of WGS based on the length of the fragments: short‐read WGS and long‐read WGS. Each has its benefits and limitations, with short‐read WGS achieving higher sensitivity and specificity at a lower expense but not being able to properly map highly repetitive areas of the DNA. Long‐read WGS is capable of correctly mapping repetitive areas of the DNA, but at an increased cost and higher error yield [8, 9]. Besides WGS, single‐cell transcriptomic and epigenetic tools such as ChIP‐seq and ATAC‐seq have also been used to identify genomic alterations in regulatory regions of the DNA [6, 10].

The increased availability of genomic data highlights the need for improved bioinformatic analysis pipelines. According to genomic experts, such as Danil C. Koboldt, the best practice for variant calling from NGS data needs rigorous preprocessing of sequencing data, implementation of multiple variant calling approaches, and a systematic filtering to remove artifacts [11]. In addition, to prevent the misrepresentation of noncoding mutations, increasing the tumor sample coverage to >80× is necessary [12, 13]. Recent WGS initiatives achieve higher sample coverage in their studies, like the Hartwig Medical Foundation (90×) and the 100.000 Genomes Cancer Programme (100×) [14, 15].

Once the somatic NCMs have been properly identified, it is necessary to conduct additional filtering to distinguish between passenger mutations and the most likely driver mutations. There are multiple methods available to prioritize mutations based on their probability of having functional effects [16]. Researchers have created algorithms capable of ranking NCMs based on potential functionality and predicting effects on chromatin conformation, such as the Activity By Contact model or DeepSEA [17, 18]. In addition, high‐throughput analysis techniques have been developed to test the functionality of thousands of noncoding mutations simultaneously. These can focus on evaluating changes in promoter–enhancer sequences, such as the Massive Parallel Reporter Assays or on changes in chromatin accessibility such as chromatin accessibility quantitative trait loci [19, 20].

### 1.2 Functional role of the noncoding genome

**Fig. 1:**
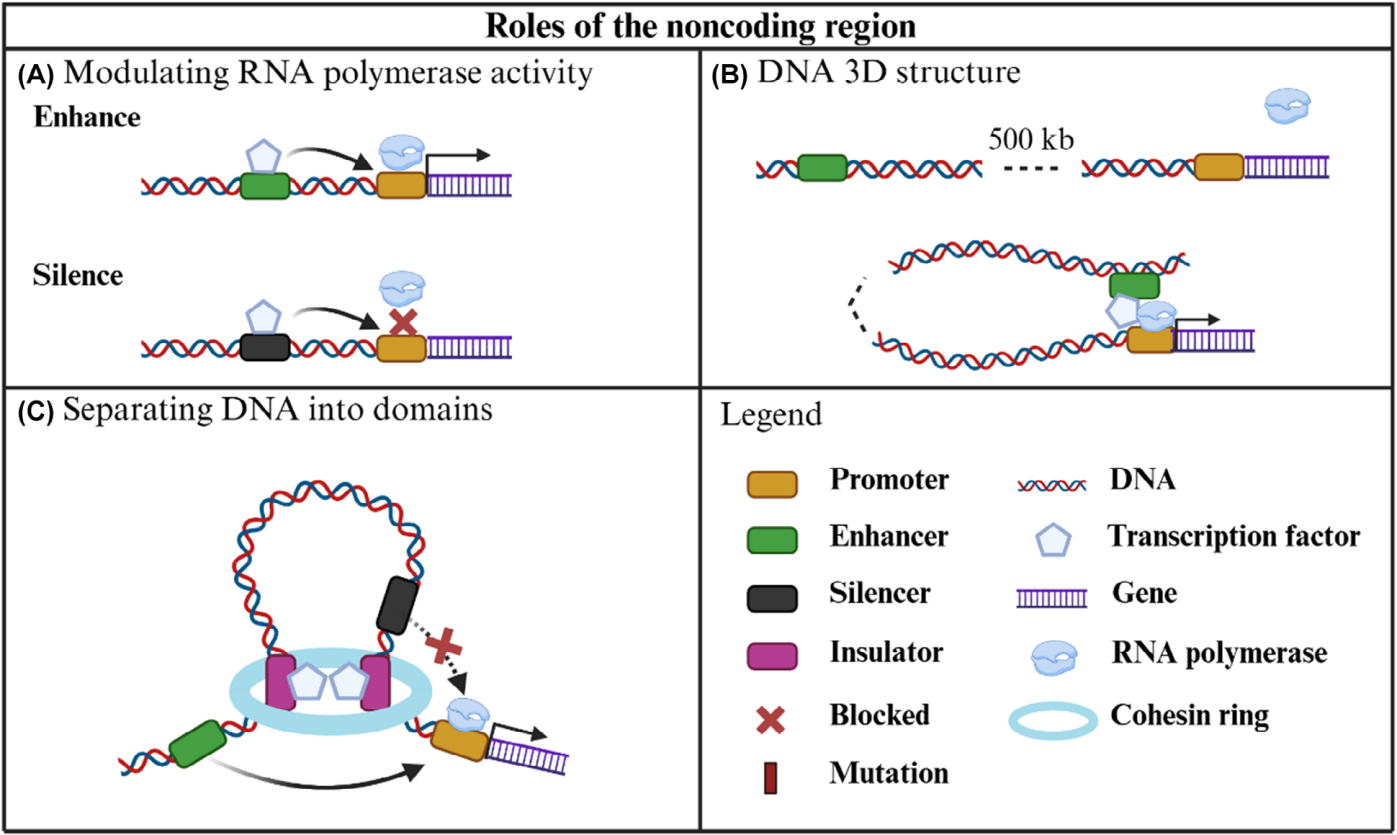
CREs are capable of affecting gene expression through different regulatory roles. (A) The transcription factors that bind to enhancers and silencers are capable of altering the RNA polymerase binding to the promoter region [6]. (B) When transcription factors bind to CREs they are capable of altering the structure of the DNA, allowing regions far apart from each other to come into 3D contact [23]. (C) CTCF and cohesin bind to insulator regions, thus creating chromatin loops that insulate the DNA inside the loop from influence from outside CREs [24]. CRE, cis‐regulatory element; CTCF, CCCTC‐binding factor. Image made using BioRender.com.

Inside the noncoding region of the genome, there are cis‐regulatory elements (CREs), which are regions of the noncoding DNA that modulate gene expression inside a chromosome by serving as binding points for transcription factors and other regulatory proteins [21]. CREs' function is tightly regulated by the availability of transcription factors that can bind, and the presence of epigenetic marks that facilitate their binding. As these conditions are dependent on both cell type and its specific developmental and physiological stage, the result is a high level of spatial and temporal regulation, providing the perfect framework to enable all possible cellular phenotypes to appear from the same DNA [22]. CREs can be classified into promoters, enhancers, silencers, and insulators; each contributing to gene expression regulation in a specific manner [21]. Promoters are the regions of the DNA where RNA polymerase binds, resulting in the initiation of gene transcription. Enhancer regions are capable of promoting gene expression by binding to transcription factors and increasing the affinity of the RNA polymerase to the promoter. On the other hand, silencer regions are capable of repressing gene expression by binding to transcription factors that block the binding of RNA polymerase to the promoter (Fig. 1A) [6]. These regions are also capable of altering the 3D structure of the DNA by the oligomerization of the transcription factors that bind to them, thus allowing CREs to influence the expression of distal genes (Fig. 1B) [23]. Finally, insulators are regions where the transcription factor CCCTC‐binding factor (CTCF) binds to and, together with the cohesin protein, forms chromatin loops that prevent CREs from interacting with DNA located outside of the looping region (Fig. 1C) [24]. This process can be clearly observed in BC, where the presence of these chromatin loops compartmentalizes the effects of the activated estrogen receptor over gene expression [25]. Moreover, changes in 3D structure caused by CTCF in BC are directly associated with increased estrogen receptor expression, as well as with increased resistance to endocrine therapies associated with increased carbonic anhydrase 2 expression [26, 27].

**Fig. 2:**
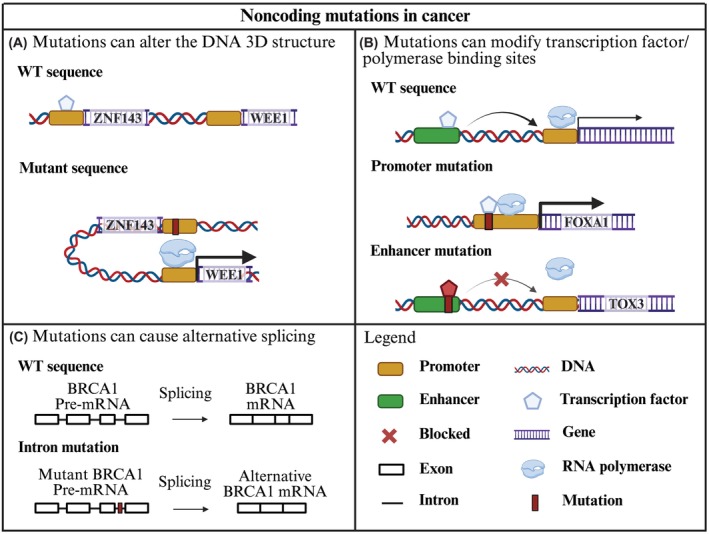
Mutations in noncoding regions can affect gene expression. (A) *ZNF143* gene promoter mutations decrease ZBTB7A transcription factor binding and promote the creation of chromatin loops with the promoters of surrounding genes (such as *WEE1*), which increases their expression [31]. (B) Mutations in enhancer and promoter regions can alter transcription factor binding and positively or negatively impact gene expression depending on which kind of transcription factor binds to the mutant region. A mutation in the promoter of *FOXA1* increases the binding of E2F family transcription factors and promotes *FOXA1* expression, while a mutation in the enhancer region of *TOX3* increases binding to the transcription repressor FOXA1 and decreases *TOX3* expression [32, 33]. (C) Mutations in the intronic region of the *BRCA1* gene can cause alternative splicing of *BRCA1's* pre‐mRNA, loss of exon 9 and creation of a truncated *BRCA1* mRNA [34]. BRCA1, breast cancer gene 1; FOXA1, forkhead box protein A1; TOX3, thymocyte selection associated high mobility group box family member 3; WEE1, Wee1‐like protein kinase; WT, wild‐type; ZBTB7A, Zinc Finger and BTB domain containing 7A; ZNF143, Zinc Finger Protein 143. Image made using BioRender.com.

According to the COSMIC database, the presence of somatic mutations in noncoding regions is three times higher than the number of somatic mutations identified in coding regions (20 million vs 6 million) [28]. In addition, genome‐wide association studies (GWAS) observed that 90% of the variance between cancer samples came from mutations in the noncoding regions of the genome [29]. Due to the important function of the noncoding genome in gene regulation, mutations in these regions can cause changes in gene expression and can be involved in cancer progression [30]. NCMs can alter the chromatin 3D structure and cause distal CREs to affect the expression of genes that would otherwise not be affected. One example observed in BC is the promoter mutation of the Zinc Finger Protein 143 gene (*ZNF143*) (chr11:9469680). This mutation promotes the binding of the Zinc finger and BTB domain‐containing protein 7A (ZBTB7A) transcription factor and increases the expression of three surrounding genes [Transmembrane protein 41B (*TMEM41B*), Importin‐7 (*IPO7*) and Wee1‐like protein kinase (*WEE1*)] by forming chromatin loops which interact with their promoters (Fig. 2A) [31]. In addition, CRE mutations can alter transcription factor binding affinity and aberrantly increase/decrease the expression of a gene. This has been observed in BC where a mutation in the promoter of Forkhead box protein A1 gene (*FOXA1*) (chr14:38064406) promotes the binding of E2F family transcription factors and increases gene expression, or with an enhancer mutation that increases the binding of transcription repressors, which results in decreased expression of the Thymocyte selection associated high mobility group box family member 3 gene (*TOX3*) (Fig. 2B) [32, 33]. Finally, NCMs located in intronic regions can result in the production of nonfunctional or truncated proteins by altering their pre‐mRNA splicing. Researchers observed this in BC samples where the presence of the c.593 + 4A>G mutation in the intronic region of the breast cancer gene 1 (*BRCA1*) gene causes alternative splicing and the omission of exon 9 in the final *BRCA1* mRNA (Fig. 2C) [34].

## 2 Noncoding mutations in promoter regions in BC patients

Research has demonstrated that there are multiple noncoding aberrations that promote BC progression and even affect response to treatment [35]. Several of these aberrations have been identified inside promoter regions (Table 1).

**Table 1:** Promoter regions with recurrent noncoding mutations in breast cancer. BC, breast cancer; BRCA1, breast cancer gene 1; E2F, E2F transcription factor family; ER, estrogen receptor; ESR2, estrogen receptor 2; FNTB, farnesyltransferase subunit β; FOXA1, forkhead box protein A1; HER2, human epidermal growth factor receptor 2; MDM2, mouse double minute 2 homolog; TNBC, triple negative breast cancer; ZBTB7A, zinc finger and BTB domain containing 7A; ZNF143, zinc finger protein 143.

Mutation in promoter region of	Function	Changes associated with BC subtype	Preclinical data	Clinical data	Role of mutations	References
FOXA1 (chr14: 38064406)	Opens chromatin facilitating ER binding	Promoter mutations observed in 2.9% of BC patients, predominantly in ER+/HER2‐ patients	Increased gene expression by facilitating E2F transcription factor binding in BC cells FOXA1 overexpression caused resistance to fulvestrant treatment in BC cells	No clinical data found	Enhances binding of E2F family transcription factors	[20, 45]
MDM2 (rs2279744)	Inhibition of p53, thus preventing apoptosis	Promoter mutation identified in 13%–33% of BC patients No subtype specific information	Increased BC risk while decreasing median overall survival in mice Increased MDM2 mRNA expression 8‐fold and protein expression 4‐fold in mice	Associated with increased BC risk in Asian women Associated with higher tumor grade	Enhances binding of Sp1	[46, 47, 48, 49]
FNTB (rs3215788, rs11623866, rs192403314)	Farnesylation of proteins	Mainly studied in TNBC	No animal model or *in vitro* studies found	rs3215788 was associated with increased histopathological grading and improved overall survival in TNBC patients rs11623866 was associated with increased tumor size, grading and improved overall survival in TNBC patients rs192403314 was associated with a higher tumor stage	rs3215788 enhances binding of GLIS3 Role not known for rs11623866 and rs192403314	[50]
ESR2 (rs2987983, rs1271572)	Nuclear receptor transcription factor.	No subtype specific information.	No animal model or *in vitro* studies found	rs2987983 is associated with increased BC risk in Caucasian women rs1271572 is associated with lower 5‐year survival and higher chance of metastasis in Chinese BC patients	rs1271572 reduces binding of Ying Yang 1 rs2987983's role not known	[51, 52, 53]
BRCA1 (rs8176318)	DNA damage repair and tumor suppressor	Observed in all BC subtypes, but associated with TNBC	Decreased gene expression in TNBC cells	TNBC patients with the 3′UTR rs8176318 mutation have a decrease in BRCA1 expression, higher risk of BC and worse prognosis in Irish women	Enhances binding of miRNAs miR‐20a‐3p and miR‐5001‐3p	[54, 55]
ZNF143 (chr11: 9482683)	Regulates DNA 3D structure	Associated with ER+ BC	Decreases luciferase expression Increases expression of surrounding genes but not ZNF143 Increases tamoxifen resistance in MCF7 cells	No clinical data found.	Decreased binding of ZBTB7A and promotes formation of chromatin loops	[20, 38, 44]

Forkhead box protein A1 (FOXA1) is a transcription factor that binds to condensed chromatin and opens it to facilitate ER binding and modulation of its transcriptional activity [36]. FOXA1 overexpression is commonly observed in ER+ BC patients, where it causes the appearance of a transcriptome profile that predicts resistance to endocrine therapy and immune checkpoint blockade treatment [37]. Through deep sequencing analysis of 360 primary BCs, FOXA1 promoter mutations were observed in 2.9% of BC patients, predominantly in ER+ BCs. Reporter assays in BC cell lines showed an increased FOXA1 expression in the presence of the promoter mutation chr14:38064406, which was mediated by an increased binding of E2F family transcription factors to the promoter [32]. Additionally, the same study observed that the FOXA1 overexpression phenotype caused by the presence of the promoter mutation caused resistance to fulvestrant treatment in BC cell lines [32, 38].

Mouse double minute 2 homolog (*MDM2*) gene codes for the E3 ubiquitin‐protein ligase, a protein involved in the inhibition of the tumor suppressor protein p53 by ubiquitinating p53 and forwarding it for proteasomal degradation. MDM2 overexpression causes an inhibition of the p53 pathway, resulting in a dysregulation of apoptosis and increased proliferation rates [39]. *In vivo* studies of the MDM2 promoter mutation rs2279744 (also known as SNP309) in transgenic mice demonstrated that it caused a significant increase in MDM2 expression and increased the risk of developing BC while decreasing overall survival [40]. A meta‐analysis evaluated the risk of developing BC based on the presence of rs2279744 between 22 764 BC patients and 22 444 healthy individuals. Their findings showed an association between the presence of the promoter mutation and increased risk of BC in Asian populations, but not in Caucasian populations [41]. Another study encompassing tumor samples from 275 BC patients, observed the rs2279744 mutation was found predominantly in those patients with higher tumor grade [42].

The farnesyltransferase subunit β (*FNTB*) gene codes for a subunit of the farnesyltransferase protein. The protein attaches a farnesyl group to the cysteine residue located in the C‐terminal tetrapeptide of key signal transduction proteins, such as the protooncogenes from the Ras family [43]. A 2022 study by Bachmann et al. [44] identified how specific mutations in the promoter of *FNTB* could be used as predictors of survival in TNBC patients. They evaluated the clinical relevance of three promoter mutations, FNTB‐173 6G > 5G (rs3215788), −609 G > C (rs11623866), and − 179 T > A (rs192403314) in 797 early breast cancer patients. Their findings identified how the presence of rs3215788 was associated with a higher tumor grading. rs11623866 was associated with a higher tumor grading and increased tumor size, and rs192403314 was related to an increased tumor stage. It was observed that the presence of rs3215788 and rs11623866 correlated with increased overall survival of TNBC patients [44].

The estrogen receptor 2 (*ESR2*) gene codes for the estrogen receptor beta protein (ESRβ), a nuclear receptor that regulates gene expression in response to estrogen [45]. An analysis of *ESR2* promoter mutations in 318 Caucasian BC patients and 318 age‐matched controls demonstrated a correlation between the presence of rs2987983 and increased risk of developing BC [46]. A study focused on evaluating the potential effects of the promoter mutation rs1271572, evaluating its presence across 873 BC patients, 645 patients with breast fibroadenoma and 700 healthy donors. rs1271572 was identified with higher recurrence in BC patients and correlated with a worse prognosis, higher chance of brain metastasis and a shorter overall survival [47].

The breast cancer gene 1 (*BRCA1*) is a tumor suppressor gene that plays a key role in the DNA repair mechanism. Germline mutations in the *BRCA1* gene are related to a high risk of breast and ovarian cancer [48]. However, recent research in the noncoding area of the genome has identified how noncoding mutations surrounding the *BRCA1* gene can also have an effect on its expression. A study focused on the *BRCA1* 3′UTR mutation rs8176318 showed how it caused a significant decrease in gene expression in TNBC cell lines by reporter assays. To confirm these *in vitro* findings, researchers obtained tumor samples from 728 BC patients and proved that the presence of rs8176318 correlated with a significant decrease in BRCA1 expression in TNBC patients. In addition, they also observed that the rs8176318 variant was more commonly identified in those BC patients with a higher tumor stage [49]. Finally, the rs8176318 variant was screened in samples from 39 BC patients and observed how its presence correlated with an increased risk of developing TNBC, suggesting that it could be used as a biomarker of TNBC risk [50].

The Zinc Finger Protein 143 (ZNF143) is a transcription factor that mediates the formation of CTCF loops to make enhancers and promoters come into 3D contact [51]. In the study by Rheinbay et al., it was identified that the promoter of ZNF143 was a hotspot for recurrent noncoding mutations in BC patients and that these mutations were found predominantly in estrogen‐positive BCs. In addition, when analyzed in reporter assays, the chr11: 9482683 mutation caused a significant decrease in luciferase expression [32]. Further studies conducted using CrisprCas9 base editing in MCF7 cells to introduce the chr11: 9482683 mutation observed that it decreased the binding of the transcription factor ZBTB7A and caused a significant increase in the expression of three surrounding genes (*TMEM41B, IPO7*, and *WEE1*), by promoting the formation of DNA loops between the mutant promoter and these genes. Interestingly, it did not cause a significant change in ZNF143 expression. Finally, the introduction of the mutation also caused a significant increase in tamoxifen resistance in MCF7 cells [31].

## 3 Noncoding mutations in enhancer regions in BC patients

In addition to promoter mutations, researchers have also identified multiple enhancer mutations associated with BC (Table 2).

**Table 2:** Enhancer regions with recurrent noncoding mutations in breast cancer. BC, breast cancer; CBP, CREB‐binding protein; c‐MYB, cellular myeloblastosis proto‐oncogene; ER, estrogen receptor; ESR1, estrogen receptor 1; FOXA1, forkhead box protein A1; HER2, human epidermal growth factor receptor 2; TNBC, triple negative breast cancer; TNFSF10, tumor necrosis factor superfamily member 10; TOX3, thymocyte selection associated high mobility group box family member 3.

Mutation in enhancer region of	Function	Changes associated with BC subtype	Preclinical data	Clinical data	Role of mutations	References
ESR1 (rs2046210, rs9383590 and rs6913578)	Nuclear receptor for estrogen	Mutated in 7% of ER+ BC patients	rs2046210 was not observed to have an effect over luciferase expression in BC cells rs6913578 caused an increase in luciferase expression in BC cells	rs2046210 is associated with increased BC risk in patients with Chinese, Japanese or European ancestry rs9383590 is associated with increased BC risk in European patients <55 years old	rs9383590 decreases binding of GATA3 rs6913578 altered DNA‐nuclear protein interaction rs2046210 known	[56, 57, 58, 59]
TOX3 (rs4784227)	Transcriptional coactivator of the histone acetylating complex p300/CBP	Associated with ER+ BC patients	Decreases TOX3 gene expression, causing an increase in cell proliferation in BC cells Alters binding of FOXA1 transcription factor in BC cell lines	Associated with increased BC risk	Increases affinity for FOXA1 and transducin‐like enhancer protein 1	[39, 60]
TNFSF10 (rs13074711)	Selectively induces apoptosis in tumor cells	Associated with TNBC	Increases TNFSF10 expression and interferon‐gamma directed apoptosis by increasing c‐MYB binding to the enhancer in BC cells.	Associated with increased risk of ER‐ BC in African American women.	Increased affinity to C‐MYB	[61, 62, 63]

The estrogen receptor 1 gene (*ESR1*) codes for the estrogen receptor protein (ER), a nuclear receptor transcription factor member of the family of estrogen receptors, like the previously described ESRβ. ER is dysregulated in approximately three out of four BC patients and it is one of the main biomarkers to stratify BC patients for treatment selection. A study from 2016 by Bailey et al. [52] identified how mutations in the enhancer region interacting with the *ESR1* gene could contribute to its dysregulation in up to 7% of ER+ BC patients. By screening the region surrounding the *ESR1* gene in 52 different ER+ BC patients, they characterized three mutations (rs2046210, rs9383590, and rs6913578) capable of modulating *ESR1* expression by altering the binding of transcription factors to the enhancers [52]. *In vitro* luciferase assays identified significant changes in expression in the presence of rs6913578 and rs9383590 but not rs2046210 in BC cell lines [53, 54]. The association of rs2046210 and BC risk was observed in both Chinese and European populations (*n* = 1554 BC vs. 1576 controls and 1590 BC vs. 1466 controls respectively), with a stronger association with ER‐ BC [55]. A separate study obtained breast samples from European (*n* = 8258), Chinese (*n* = 18 414), and Japanese (*n* = 3142) patients and observed that the presence of rs2046210 correlated with increased BC risk in these populations [53]. A final study including samples from 409 BC patients and 422 female controls observed no correlation between BC risk and rs9383590, theorized to be due to its low recurrence (5/409) [54].

The exact function of Thymocyte selection associated high mobility group box family member 3 (TOX3) protein has not been properly characterized yet, though a study from 2011 suggests that it is involved in the activation of the histone acetylating complex p300/CREB‐binding protein (CBP), thus promoting gene expression [56]. A multistage GWAS including over 28 000 cases and controls from Chinese and European‐American women was conducted to identify susceptibility loci for BC. rs4784227, a mutation in an enhancer region 18 kb from the *TOX3* gene, was found to increase BC risk and be associated with ER+ BC [57]. They also conducted luciferase assays in BC cells to evaluate its functional effects and observed that it caused a significant decrease in luciferase expression [57]. These results correlate with an additional study conducted by Cowper‐Sallari et al. [33], where rs4784227 increased the affinity for the FOXA1 protein and the transcription repressor transducin‐like enhancer protein 1, thus decreasing *TOX3* expression. Mimicking the rs4784227 phenotype by silencing *TOX3* in BC cell lines, they observed a significant increase in cell proliferation.

The tumor necrosis factor superfamily member 10 (*TNFSF10*) gene codes for a protein called tumor necrosis factor‐related apoptosis‐inducing ligand (TRAIL) and it selectively induces apoptosis in tumor cells without affecting normal cells [58]. A population study including 1984 African American BC patients and 2939 controls identified the rs13074711 mutation in an enhancer region 26.5‐kb upstream of TNFSF10, and observed that the enhancer mutation was associated with an increased risk of ER‐ BC [59]. *In vitro* studies aiming to identify the functional impact of rs13074711 in TNBC cell lines observed that it increased the affinity of the transcription factor MYB proto‐oncogene (C‐MYB) for the enhancer region, which resulted in an upregulation of TRAIL expression and an increased induction of apoptosis [60].

## 4 Genetic alterations in lncRNAs in BC patients

**Fig. 3:**
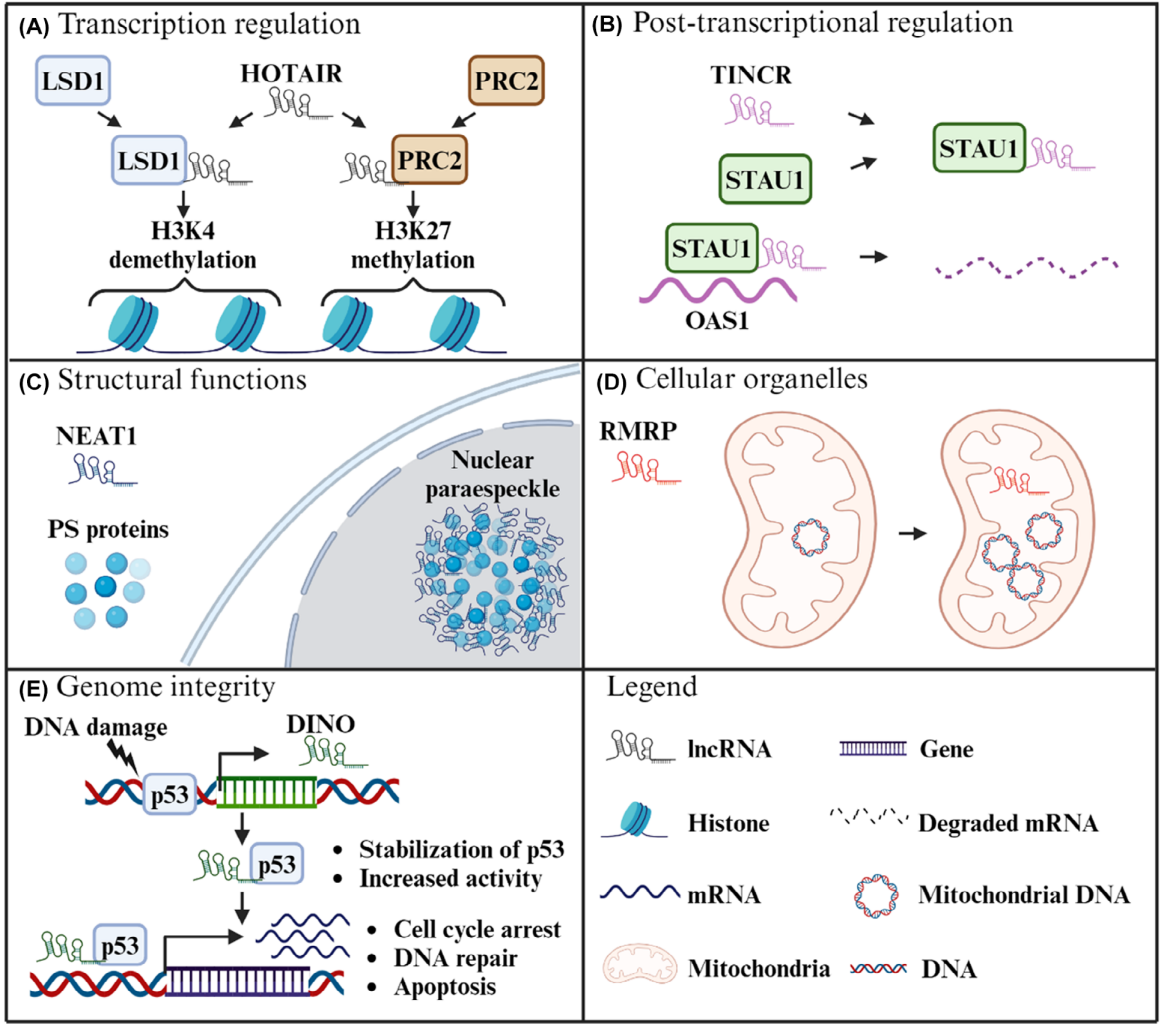
Different roles of lncRNAs. (A) *HOTAIR* regulates transcription by binding to histone methylation/demethylation proteins (LSD1 and PRC2) and guiding them to their targets [62]. (B) *TINCR* regulates gene expression at a post‐transcriptional level by binding to the mRNA degrading protein STAU1 and guiding it to its targets, like *OAS1's* mRNA [63]. (C) *NEAT1* is the structural basis of nuclear paraspeckles, nuclear organelles created by paraspeckle proteins interwoven with *NEAT1*, which are involved in gene regulation by sequestration of regulatory RNAs and splicing factors [64]. (D) *RMRP* is involved in cell cycle progression by promoting the replication of mitochondrial DNA [65]. (E). *DINO* transcription is activated by the tumor suppressor p53 in response to DNA damage. Binding of *DINO* to p53 creates a forward loop increasing its activity [66]. DINO, damage induced long noncoding RNA; H3K27, histone 3 lysine 27; H3K4, histone 3 lysine 4; HOTAIR, HOX transcript antisense intergenic RNA; lncRNA, long‐noncoding RNA; LSD1, lysine‐specific histone demethylase 1A; NEAT1, nuclear paraspeckle assembly transcript 1; OAS1, 2′‐5′‐oligoadenylate synthetase 1 mRNA; p53, cellular tumor antigen p53; PRC2, polycomb repressive complex 2; PS, paraspeckles; RMRP, RNA component of mitochondrial RNA processing endoribonuclease; STAU1, Staufen double‐stranded RNA binding protein 1; TINCR, tissue differentiation‐inducing non‐protein coding RNA. Image made using BioRender.com.

Besides CREs, the noncoding DNA also contains RNA coding genes. They are considered parts of the noncoding DNA because the RNA that these genes code for is not translated into proteins. Noncoding RNAs with over 200 bps are considered long noncoding RNAs (lncRNA) and they are involved in the regulation of several key cellular functions [61]. Their main functions range from regulating gene expression at the epigenetic level by guiding histone methylation/demethylation to specific regions (Fig. 3A) [62]; disrupting RNA translation by guiding RNA degrading proteins to mRNAs (Fig. 3B) [63]; forming nuclear structures of RNA binding proteins to trap lncRNAs and prevent their function (Fig. 3C) [64]; promoting mitochondrial DNA replication during mitosis (Fig. 3D) [65]; and preserving genomic integrity through stabilizing p53 upon DNA damage and guiding it to its target regions (Fig. 3E) [66]. There are several lncRNAs that have shown to play a role in BC (Table 3).

**Table 3:** LncRNAs with demonstrated effect over breast cancer. BC, breast cancer; DANCR, differentiation antagonizing non‐protein coding RNA; ER, estrogen receptor; H19, lncRNA H19; HER2, human epidermal growth factor receptor 2; HOTAIR, HOX transcript antisense intergenic RNA; MALAT1, metastasis associated lung adenocarcinoma transcript 1; NEAT1, nuclear paraspeckle assembly transcript 1; RMRP, RNA component of mitochondrial RNA processing endoribonuclease; TINCR, tissue differentiation‐inducing non‐protein coding RNA; TNBC, triple negative breast cancer.

Lnc RNA	Function	Changes associated with BC subtype	Preclinical data	Clinical data	References
*HOTAIR*	Epigenetic silencing	Associated with TNBC	Inhibition reduces proliferation and migration of BC cells and sensitizes them to tamoxifen treatment Inhibition impairs metastasis and invasion in mice	Overexpression associated with worse survival and higher metastasis rate in ER+ BC patients	[46, 51, 52, 53]
*TINCR*	Post transcriptional regulation of mRNA	Overexpressed in HER2+ BC patients	Knockdown decreases tumor growth in mice Knockdown decreased proliferation and migration of BC cells	Overexpression associated with decrease overall survival	[47, 54, 55]
*NEAT1*	Formation of nuclear paraspeckles	Mutation identified in all BC subtypes, higher association with TNBC	Inhibition causes cell cycle arrest and apoptosis in BC cells Silencing inhibits tumor growth and metastasis in mice models and sensitizes them to cisplatin treatment	Upregulated BC patients High expression associated with cancer progression and decreased sensitivity to chemotherapy	[48, 56, 57]
*RMRP*	Mitochondrial replication	Promoter mutated in 2.5% of BC but no subtype specific information	Knockout causes a decrease in the growth and migration capabilities of BC cells, while overexpression enhances them	Upregulated in BC patients High RMRP expression associated with lower overall survival	[19, 49, 58]
*DANCR*	Negative regulator of cell differentiation.	Mutation identified in all BC subtypes, higher association with TNBC	Knockdown reduces tumor growth in mice Inhibition reduces cell proliferation and migration in BC cells Knockdown reduces inflammation in BC cells	Upregulated in BC patients High expression correlates with decreased overall survival and increased BC risk	[59, 60, 61, 62, 63]
*H19*	microRNA sponge	Overexpressed in 70% of BC patients. Associated with HER2‐ BCs	Knockdown correlates with decrease in colony formation and migration in BC cells Inhibition decreases tumor size, invasion and metastasis in mice	High levels correlate with BC and higher degree of malignancy	[64, 65, 66]
*MALAT1*	Gene transcription and pre‐mRNA splicing	Duplicated in 12% of TNBCs	Loss reduces migration and metastasis in mice Knockdown reduces migration and tumor phenotype of BC cells	Higher levels associated with a decrease in survival of ER‐ BC patients without lymph node involvement	[67, 68, 69, 70, 71]

The HOX transcript antisense intergenic RNA (*HOTAIR*) is a transacting lncRNA, key for the correct epigenetic regulation of gene expression (Fig. 3A) [62]. *In vitro* studies demonstrated how *HOTAIR* knockdown resensitized BC cell lines to tamoxifen, reducing their proliferative rate [67]. Moreover, *in vivo* studies demonstrated how the inhibition of *HOTAIR* expression results in a significant reduction in the size of breast tumors transplanted to mouse models [68]. A study including tissues from 164 primary BC tumors observed that overexpression of *HOTAIR* was significantly associated with a worse prognosis and a decrease in metastasis‐free survival. The association was more pronounced in ER‐ BC patient samples [69]. Finally, overexpression of *HOTAIR* is associated with decreased treatment response to tamoxifen in ER+ BC patients [67].

Tissue differentiation‐inducing nonprotein coding RNA (*TINCR*) is a lncRNA involved in post‐transcriptional regulation of mRNAs. *TINCR* overexpression causes an increase in cell survival by promoting the degradation of the mRNA of 2′‐5′‐oligoadenylate synthetase 1 (OAS1), a protein involved in antiviral response and apoptosis (Fig. 3B) [63]. Preclinical studies highlighted how the inhibition of *TINCR* reduced the malignant progression and migration capabilities of BC cells, and decreased tumor size in mouse xenograft models [70]. An *in silico* analysis of expression data from 1088 BC patients identified an overexpression of *TINCR* compared to normal tissues. By measuring *TINCR* expression in samples from 33 additional BC donors, overexpression was found in HER2+ BC patients and was associated with a decrease in overall survival [71].

Nuclear paraspeckle assembly transcript 1 (*NEAT1*) is a lncRNA found in the nucleus of mammalian cells. Its main function is to construct paraspeckles, protein‐rich nuclear organelles built around a specific lncRNA scaffold, which directly affect expression of the surrounding genes (Fig. 3C) [64]. An analysis of sequencing data surrounding the *NEAT1* gene identified a hotspot of mutations in the *NEAT1* promoter capable of causing a decrease in its expression. *NEAT1* silencing in BC cell lines causes a significant decrease in cell proliferation and migration [72]. In addition, an *in vivo* xenograft tumor model identified that *NEAT1* silencing caused a significant decrease in tumor size and further sensitized the BC cells to cisplatin treatment [73]. When *NEAT1* expression was compared between samples from 179 BC patients and 192 normal controls, a significantly higher level of *NEAT1* was observed in BC patients. These results also showed that this increased expression was more prominent in TNBC patient's samples [73]. A different analysis comparing clinical data from 40 BC patients, saw a correlation between higher *NEAT1* expression and a shorter overall survival [72].

RNA component of mitochondrial RNA processing endoribonuclease (*RMRP*) is a lncRNA involved in cell cycle progression and mitochondrial DNA replication (Fig. 3D) [65]. A deep sequencing study of 360 primary BCs identified that 2.5% of the patients had mutations in the promoter of *RMRP*. Two mutations (35658033 G>A and 35658025 G>C) caused a significant increase in expression through luciferase assays in BC cells [32]. An *in vitro* analysis of the effects of *RMRP* silencing in BC cell lines observed a significant decrease in cell growth and migration, mediated by a decrease in RAC‐alpha serine/threonine‐protein kinase (AKT) activation. *RMRP* expression promotes AKT activation through the sequestering of the micro‐RNA miR‐206, preventing it from blocking AKT mRNA translation [74]. When comparing *RMRP* expression between BC tumor samples and normal breast samples (*n* = 165 vs. 33), an increase in *RMRP* was observed in BC samples. This increase correlated with a decrease in overall survival [74].

Differentiation antagonizing nonprotein coding RNA (*DANCR*) is an oncogenic lncRNA that acts as a negative regulator of cell differentiation and is involved with multiple cancers [75]. *In vitro* assays showed how knockdown of *DANCR* caused a significant decrease in proliferation and invasion of TNBC cell lines [76]. Moreover, *DANCR* knockdown was also observed to inhibit the stem cell phenotype and reduce the production of inflammatory cytokines of BC cells [77]. Xenograft mice studies demonstrated that *DANCR* silencing significantly decreased tumor size, as well as migration [78]. Analyzing gene expression data from 1104 BC patient samples of The Cancer Genome Atlas database, *DANCR* is significantly overexpressed in BC patients, especially in TNBC. In addition, *DANCR* expression was also related with a significant decrease in overall survival of BC patients [79].

lncRNA H19 (*H19*) regulates gene expression by acting as a sponge for microRNAs and blocking their regulatory effects. *In vitro* studies showed that silencing of the *H19* gene resulted in significant decreases in the invasion and colony‐forming capabilities of BC cell lines [80]. In addition, animal studies demonstrated how knockdown of *H19* resulted in a significant decrease in primary tumor size, as well as a decrease in lymph node metastasis in mice [80]. A study analyzing *H19* expression in 102 BCs found an overexpression in 72.5% of cases. Overexpression of *H19* was frequently observed in ER+ BC patients [81]. A more recent study including 60 BC patient samples, identified that *H19* expression was also increased in TNBC patients [80]. An additional study including 79 tissue samples from BC patients observed how elevated *H19* levels correlated with a higher risk of BC and an increased degree of malignancy [82].

Metastasis‐associated lung adenocarcinoma transcript 1 (*MALAT1*) is a highly conserved lncRNA that has a regulatory effect on gene expression at transcriptional and post‐transcriptional levels by modulating the phosphorylation of the serine‐arginine rich splicing factor [83]. *In vitro* studies identified that knockdown of the *MALAT1* gene significantly reduced the migration and proliferation of BC cell lines by causing cell cycle arrest [84]. *In vivo, MALAT1* inhibition significantly reduced the capabilities of MDA‐MB‐231 BC cells to colonize and develop metastasis in immunodeficient mice [85]. A study analyzing tandem duplications in BC patients' WGS data (*n* = 1992) found that 12% of TNBCs have a duplication of the *MALAT1* gene, causing its overexpression [86]. Overexpression of *MALAT1* has been associated with a decrease in the survival of ER‐ BC patients without lymph node involvement [87].

## 5 Conclusion and future perspectives: The critical need for functional validation

Most of the research regarding the evaluation of new cancer drivers and biomarkers of response to treatment focuses on the identification of novel mutations inside of genes (coding mutations) [88]. However, the continuous advances in sequencing technology and computational capabilities have provided a new perspective over the human genome that encouraged scientists to further the study of the noncoding regions and their regulatory capabilities over gene expression [89]. Every year, there are more studies highlighting the importance of the noncoding regions of the genome and how they influence cancer progression [90]. However, there exist bottlenecks that limit the application of many of the findings identified in the laboratory to the clinic.

First, despite the emergence of multiple high‐throughput analysis techniques for the analysis of NCMs, they are only able to provide a raw value of the potential functional effect of these NCMs without taking into account the model or the genomic context [91, 92].

Second, as observed with the NCMs described in this review, most functional studies have focused on evaluating functionality via either reporter assays, correlative studies with clinical samples, chromatin immunoprecipitations or knockdown of the mutation site [6, 32]. These techniques, albeit informative, fail to represent the complex regulation of the noncoding genome and the exact effects of the NCMs over the cell's phenotype [16]. Nowadays, the most reliable method to identify the function of noncoding mutations *in vitro* requires the use of CRISPR‐Cas9 gene editing technologies to introduce the desired NCM into a context‐appropriate model [93, 94]. However, limitations to this approach include it being very time‐consuming, the chromatin accessibility in the noncoding region varying greatly across cell models, and the high degree of redundancy in the noncoding genome limiting Cas9 delivery to the desired edit site. Recent advances aimed to improve the efficacy of the gene edit while minimizing the appearance of additional unwanted edits. One such gene editing method uses a mutant Cas9 protein where it loses its double‐strand break capabilities in favor of a single‐strand nickase activity. Two Cas9‐nickases must attach to the desired edit region, to create a double‐strand break, therefore significantly decreasing the ratio of off‐target effects [6, 95]. Later *in vitro* testing of the NCMs effects should be conducted by creating 3D culture organoids using the CRISPR‐edited cell lines and transplanting them into animal models [6, 16].

Third, the presence and activity of NCMs are very dependent on the cancer type being studied. The majority of the NCMs described in this review have only been observed in BC patients, with only the promoter mutations rs2279744 (*MDM2*) and rs2987983 (*ESR2*) being associated with increased risk of other cancers (leukemia and prostate cancer respectively) [96, 97]. The limited availability of data for these NCMs in different cancers could be attributed to the existence of tissue‐specific CREs, as cancers originating from different tissues have a different map of the active noncoding genome. This level of specificity makes it difficult to compare different cancer types and limits the one‐fits‐all approach to studying NCMs [98, 99]. In comparison, lncRNAs are more conserved across different tissues [61]. All of the lncRNAs included in this review are associated with multiple cancer types. For example, *HOTAIR* overexpression is not solely associated with poor prognosis and metastasis in BC, but also in colorectal, endometrial and lung cancer [62]. In addition, *TINCR* overexpression is associated with a decrease in overall survival in BC, but also in esophageal and liver cancer [100]. The fact that lncRNAs are more widespread and easier to measure has resulted in increased research focus over the past few years [101, 102].

Proper functional profiling of novel NCMs is crucial before they can be considered for clinical evaluation. By understanding the mechanisms through which they dysregulate the tumor's phenotype, researchers will be able to identify drugs to treat these NCMs and conduct clinical testing in context‐appropriate models.

This testing should focus on evaluating NCMs' performance and clinical utility compared to already established biomarkers, observing correlation with patient response and pathogenicity [103]. After the validation of novel NCMs as biomarkers, their application in the clinical setting would be relatively simple, as routinely used diagnostic tools to detect mutational status are available, for example, real‐time PCR‐based detection of *TERT* promoter mutations in glioblastomas [90].

## Conflict of interest

The authors declare no conflict of interest.

## Author contributions

ACS, AE, and JC were involved in conceptualization. ACS and AM‐M were involved in writing of manuscript. ACS was involved in research. AE and JC were involved in review of manuscript
